# Intraoperative Clonidine in Spine Surgery: A Randomised Controlled Trial

**DOI:** 10.1111/aas.70048

**Published:** 2025-05-07

**Authors:** Stine Birkebæk, Niels Juul, Mikkel Mylius Rasmussen, Peter Gaarsdal Uhrbrand, Lone Nikolajsen

**Affiliations:** ^1^ Department of Clinical Medicine Aarhus University Aarhus Denmark; ^2^ Department of Anaesthesiology and Intensive Care Aarhus University Hospital Aarhus Denmark; ^3^ Department of Neurosurgery Aarhus University Hospital Aarhus Denmark

**Keywords:** intraoperative clonidine, postoperative pain, spine surgery

## Abstract

Patients undergoing spine surgery often experience post‐operative pain. In this context, clonidine, an alpha‐2 agonist, may be relevant due to its analgesic properties. We conducted a randomised, double‐blinded, placebo‐controlled trial to evaluate the effect of a single dose of intraoperative intravenous clonidine on post‐operative opioid consumption, pain intensity and side effects. Patients undergoing spine surgery at Aarhus University Hospital, Denmark, were randomised to receive intraoperative clonidine (3 μg/kg) or placebo. The primary outcome was opioid consumption within the first 3 h after surgery. Secondary outcomes included opioid consumption within the first 6 h, pain intensity at rest and during coughing, post‐operative nausea and vomiting (PONV), and sedation in the post‐anaesthesia care unit (PACU). Additional outcomes included time to discharge from the PACU, length of hospital stay and daily opioid consumption after 1 month. Data from 120 patients (49 females, 71 males, mean age 65 ± 14 years) were available for analysis; 61 received clonidine and 59 received placebo. Post‐operative intravenous morphine equivalents within 3 h were similar in the clonidine group 5 mg (0–15) and the placebo group 10 mg (0–15) (*p* = 0.58). Pain intensity at rest was 4 (0–5.5) in the clonidine group and 3 (0–5) in the placebo group upon arrival at the PACU (*p* = 0.20). No differences were observed between the clonidine and placebo groups regarding any secondary outcomes, except for hypotension, which was more frequent in the clonidine group (24 vs. 13 patients). A single dose of intraoperative clonidine did not reduce post‐operative opioid consumption or pain intensity in patients undergoing spine surgery.

## 1 Introduction

Despite advancements in pain management strategies, many patients still suffer from post‐operative pain, reducing patient satisfaction, prolonging hospital stays and increasing the risk of persistent pain [1, 2, 3, 4]. Patients undergoing spine surgery are particularly prone to post‐operative pain and prolonged opioid use [1, 5]. Multimodal pain management is now the standard for post‐operative care, yet opioids remain the primary choice for moderate to severe pain. Although effective, opioids often cause side effects such as sedation, nausea, vomiting, constipation and carry a risk of long‐term use [6, 7]. Therefore, alternative analgesics are required to improve multimodal pain management for surgical patients.

In this context, clonidine emerges as a valuable component of multimodal pain management due to its analgesic properties. First synthesised in 1962 and originally commercialised as an antihypertensive drug [8], clonidine is now used for various clinical purposes, including managing post‐operative pain and alleviating opioid withdrawal symptoms. Clonidine exerts its effects by binding to alpha‐2 adrenergic receptors, which are widely distributed throughout the body. Its analgesic properties are thought to be mediated by receptor binding at both spinal and supraspinal levels [9, 10]. Following intravenous (IV) administration, clonidine reaches peak plasma concentration levels within 10 min and has an elimination half‐life of 6–23 h [10, 11, 12, 13]. Side effects include bradycardia, hypotension and sedation [8, 10, 11, 14].

Previous studies have suggested that perioperative IV clonidine reduces post‐operative pain intensity and opioid consumption [12, 13, 15, 16]. However, most of these studies were published several years ago and exhibit limitations related to design and sample size. In recent years, only a few randomized controlled trials (RCTs) on the perioperative use of clonidine have been published. However, these studies compare IV clonidine with other pharmacological agents, such as dexmedetomidine, ketamine and magnesium sulfate, or use other routes of administration, including intraperitoneal and intrathecal delivery [17, 18, 19, 20, 21]. Clonidine is widely used in clinical settings in Scandinavian countries, but a knowledge gap remains regarding its intraoperative use for post‐operative pain management. Therefore, this study aimed to evaluate the analgesic effect of a single dose of intraoperative IV clonidine in patients undergoing spine surgery.

## 2 Methods

### 2.1 Study Design and Approvals

This was a single‐centre, randomised, double‐blinded, placebo‐controlled trial. It was conducted in accordance with the Declaration of Helsinki and guidelines for good clinical practice (GCP) and monitored by the GCP unit at Aarhus University Hospital, Aarhus, Denmark. The study protocol was approved by the Medical Research Ethics Committees, the Danish Medicines Agency (DKMA) (CTIS‐number 2023‐505969‐80‐00) and the Danish Data Protection Agency (ID 1‐16‐02‐286‐23). Before enrolling the first patient, the study was registered at Clinical Trials Information System (CTIS‐number 2023‐505969‐80‐00), and the protocol was published at the initial trial stage for clarity [22].

### 2.2 Patients

Patients scheduled for surgical treatment of degenerative spine disease, that is, spinal fusion, spinal decompression or both [23, 24], at the Department of Neurosurgery, Aarhus University Hospital, Denmark, were eligible for participation. The exclusion criteria were: age < 18 years, American Society of Anaesthesiologists (ASA) Physical Status IV or V, allergy to clonidine, inability to provide informed consent, severe renal insufficiency (estimated glomerular filtration rate < 30), severe bradyarrhythmia, severe ischaemic heart disease, severe congestive heart failure (ejection fraction < 30%), disseminated cancer disease, pregnancy/lactation and/or planned treatment with epidural analgesia, methadone, or ketamine in the perioperative period. Oral and written informed consent was obtained from each patient before surgery. Patient screening and recruitment were initiated in September 2023; the last patient was included in March 2024.

### 2.3 Randomisation, Study Drug and Blinding

Patients were randomised in a 1:1 ratio to receive a single dose of intraoperative IV clonidine (3 μg/kg) or placebo containing isotonic saline. Opioid users (patients using opioids daily for at least 14 days leading up to surgery) and non‐users were randomised separately. The pharmacy generated the randomisation list in blocks of four and six patients.

Study drugs were delivered in sealed kits with identical outer appearances. Each kit was assigned a consecutive randomisation number and contained either vials of clonidine (Clotaxip, 2 mL × 150 μg/mL) or isotonic saline (NaCl 10 mL × 9 mg/mL, Fres. Kabi) and one bag of 100 mL isotonic saline. Patients in the clonidine group received 3 μg/kg, calculated using actual body weight for body mass index (BMI) < 25 or ideal body weight (height—100 for men, height—105 for women) for BMI > 25. The study drug was prepared by a nurse not involved in the study, who mixed either clonidine or isotonic saline from the vials into a 100 mL bag of isotonic saline and labelled it with the randomisation number. The study drug was then administered to patients by the anaesthetic staff immediately after intubation. Researchers, healthcare professionals and patients were blinded to the treatment allocation. The treatment allocation was stored in sealed opaque envelopes for emergency unblinding and remained concealed until statistical analyses and the first draft of the manuscript were completed.

### 2.4 Anaesthesia and Post‐Operative Pain Management

Surgery and anaesthesia followed standard procedures at our institution.

Patients underwent spine surgery in the prone position under general anaesthesia induced and maintained with propofol and remifentanil. The study drug (clonidine or placebo) was administered via infusion over 5–10 min immediately after intubation. Noradrenaline (10 μg/mL) was infused at a rate of 0.02–0.2 μg/kg/min in all patients. Hypotension was managed with fluids and/or vasopressors, and bradycardia with atropine at the anaesthetist's discretion. In this study, both conditions were defined by the anaesthetist's assessment of treatment necessity. Intraoperative analgesia included a single dose of paracetamol (1000 mg), fentanyl (100 μg) and ketorolac (30 mg) if not contraindicated, and morphine (0.1–0.2 mg/kg) or oxycodone (0.1–0.15 mg/kg). Bupivacaine with adrenaline (2.5 mg/mL and 5 μg/mL) was infiltrated into the skin by the surgeon before closure. To prevent post‐operative nausea and vomiting (PONV), patients received dexamethasone (4 mg) and ondansetron (4 mg). In the post‐anaesthesia care unit (PACU), post‐operative pain was managed according to pain intensity measured using a numeric rating scale (NRS, 0–10, where 0 = no pain, and 10 = severe pain). For patients with an NRS ≥ 7 at rest, a combination of IV alfentanil (0.25–0.5 mg) and morphine (0.1 mg/kg) or fentanyl alone (1 μg/kg) was administered. For NRS 4–6, IV morphine (0.05–0.1 mg/kg) was administered until NRS ≤ 3. If morphine was not tolerated due to severe side effects, IV oxycodone was administered. All opioids were registered in the electronic patient journal.

### 2.5 Outcomes

The primary outcome was opioid consumption measured as cumulative IV morphine equivalents in milligrams (mg) within the first 3 h after arrival to the PACU.

Secondary outcomes related to analgesic efficacy were opioid consumption within the first 6 h after arrival to the PACU and pain intensity at rest and during coughing measured on the NRS (0–10) at 0, 30, 60, 90 and 120 min or at discharge from the PACU if discharged before 120 min. Secondary outcomes related to side effects included PONV and sedation levels that were measured using the Ramsay Sedation Scale (1–6, where 1 = agitated, 2 = tranquil, 3–6 = increasing level of sedation). Both PONV and sedation levels were assessed at 0, 60 and 120 min or at discharge from the PACU. Additional outcomes included time to discharge from the PACU, length of hospital stay and daily opioid consumption after 1 month.

Patients reported the average intensity of their back pain and radicular pain on the NRS (0–10) during the week before surgery and their pain treatment used in the 24 h before surgery (Table 1). Post‐operative opioid consumption was retrieved from the electronic patient journal and was converted to IV morphine equivalents: conversion ratios were oxycodone (1.3:1), fentanyl (100:1) and alfentanil (10:1) [25]. Additional data were collected on a paper data sheet by a nurse from the PACU, including pain intensity at rest and during coughing (NRS, 0–10) and presence of PONV (yes/no), and level of sedation. One month after surgery, patients completed a questionnaire, reporting their consumption of analgesics over the previous 24 h. As clonidine is commonly used for managing opioid withdrawal symptoms and assisting with opioid tapering, we also measured opioid consumption after 1 month.

**TABLE 1:** Baseline characteristics.

	Clonidine (*n* = 61)	Placebo (*n* = 59)
Opioid‐user (*n* = 31) (*n*) (%)	15 (25)	16 (27)
ASA physical status (*n*) (%)		
ASA 1	8 (13)	6 (10)
ASA 2	36 (59)	30 (51)
ASA 3	17 (28)	23 (39)
Sex (*n*) (%)		
Female	27 (44)	22 (37)
Age (years) (IQR)	70 (56–77)	63 (57–76)
BMI (SD)	29 (± 6)	29 (± 6)
Smoking (*n*) (%)	8 (13)	17 (29)
Alcohol consumption (*n*) (%)		
0 items	14 (23)	10 (17)
1–21 items	46 (75)	44 (75)
> 21 items	1 (2)	5 (8)
Previous spine surgery (*n*) (%)	24 (39)	16 (27)
1–2 surgery/surgeries	21 (34)	11 (19)
> Surgeries	3 (5)	5 (8)
Preoperative pain intensity (NRS) (IQR)		
Back pain at rest	3 (1–5)	3 (1–5)
Back pain during movement	6 (4–8)	6 (4–8)
Radicular pain at rest	4 (2–6)	5 (2–7)
Treatment with opioids prior to surgery (*n*) (%)	16 (26)	18 (31)
Oral morphine equivalents in mg (IQR)	17.5 (10–42.5)	20 (20–40)
Pain treatment prior to surgery (*n*) (%)		
Neuropathic pain treatment	26 (43)	22 (37)
Paracetamol and/or NSAIDs	44 (72)	42 (71)
Medical treatment for anxiety/depression (*n*) (%)		
Yes	2 (3)	7 (12)

*Note:* Data are presented as numbers (*n*) (%) of patients, or as means with standard deviations (SD) or medians with interquartile ranges (IQR). Neuropathic pain treatment includes gabapentin, pregabalin, TCAs, and duloxetine.

### 2.6 Sample Size

The sample size calculation was based on the literature [12, 13, 15, 16] and a pre‐trial audit at our department showing that spine surgery patients received between 15 and 45 mg of IV morphine equivalents within the first 3 h after surgery (primary outcome). We expected that a single dose of 3 μg/kg clonidine would reduce the mean IV morphine consumption by 10 mg, with a standard deviation of 15 mg. To achieve a significance level of 0.05 (*α* = 0.05) and a power of 90% (*β* = 0.1), 47 patients in each arm were required. To account for dropout, we included 60 patients in each group, achieving a total study population of 120 patients.

### 2.7 Statistical Analysis

All statistical analyses were conducted using Stata software version STATA/SE 18.0 (StataCorp, College Station, Texas, USA). Numerical data were reported as means with standard deviations (SD). Non‐normally distributed numerical data (3 and 6‐h opioid consumption, time to discharge, length of hospital stay) and ordinal data (pain intensity) were presented as medians with interquartile ranges (IQR) and compared using the Mann–Whitney *U* test. Categorical data (nausea/vomiting, sedation) were reported as numbers (%) and compared using the *X* ^2^ test or Fisher's exact test as appropriate. Two‐sided *p* values below 0.05 were considered significant. Data were collected and managed using the REDCap electronic data capture tools hosted at Aarhus University, Denmark [26].

## 3 Results

### 3.1 Patients

**FIGURE 1:**
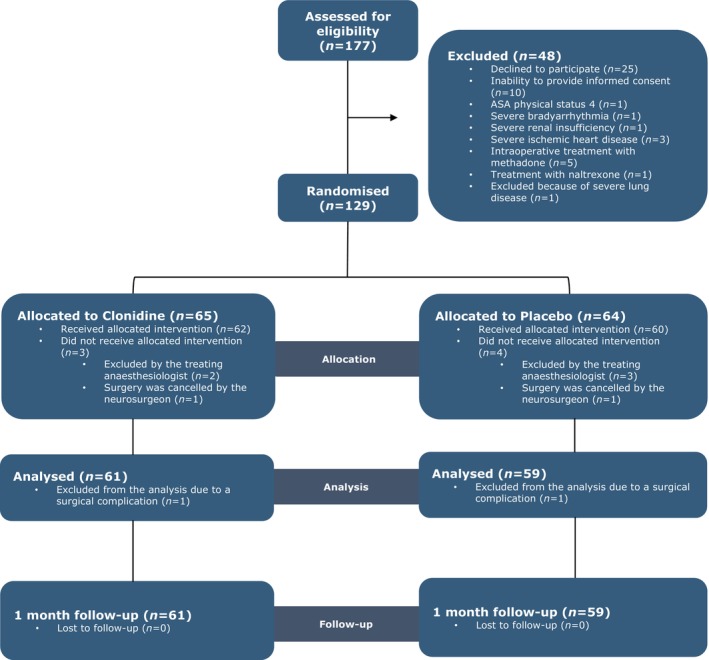
Consort flow diagram. In total, 9 patients were excluded after randomisation. Of these, 7 were excluded before receiving the study drug: 5 due to bradycardia and/or hypotension following anaesthesia induction, as determined by the treating anaesthetist, and 2 due to surgery cancellation. An additional 2 patients were excluded after receiving the study drug due to surgical complications: 1 patient in the clonidine group experienced major bleeding during surgery and required admission to the intensive care unit (ICU), and 1 patient in the placebo group developed radicular pain and decreased strength, requiring an urgent MRI scan. For both patients, it was impossible to achieve any primary or secondary outcomes.

We enrolled 129 patients, assigning 65 to clonidine and 64 to placebo (Figure 1). Of these, 122 patients received the study drug. Two patients were excluded after administration due to surgical complications: one patient in the clonidine group (major bleeding) and one patient in the placebo group (radicular pain and decreased strength in the right leg after surgery). Thus, data from 120 patients (61 clonidine, 59 placebo) were included in the final analysis. Baseline characteristics were comparable between groups (Table 1), except for a non‐significant larger proportion of patients with previous spine surgery in the clonidine group, and no differences were observed in intraoperative characteristics (Table 2).

**TABLE 2:** Intraoperative data.

	Clonidine (*n* = 60)	Placebo (*n* = 59)
Type of spine surgery (*n*) (%)		
Decompression	52 (85)	49 (83)
Spinal fusion	9 (15)	10 (17)
Spine levels (*n*) (%)		
1 level	48 (79)	46 (78)
2 or more levels	13 (21)	13 (22)
Surgery duration (min) (SD)	92 (±41)	105 (±51)
Time from surgery end to extubation (min) (SD)	13 (±8)	13 (±7)
Total propofol dose (μg) (IQR)	892 (640–1130)	996 (684–1210)
Total remifentanil dose (μg) (IQR)	3721 (2718–5508)	4345 (2759–5445)
Total fentanyl dose (μg) (IQR)	100 (100–100)	100 (100–100)
Bleeding (ml) (IQR)	150 (100–250)	200 (50–400)

*Note:* Data are presented as numbers (*n*) (%) of patients, or as means with standard deviations (SD) or medians with interquartile ranges (IQR). The anaesthesia journal was missing for one patient in the clonidine group (*n* = 60). Anaesthesia was maintained with sevoflurane for two patients in the placebo group as anaesthesia with propofol and remifentanil was not possible for these patients due to lung and cardiac disease. These two patients were not included in the analysis of propofol and remifentanil dosage (*n* = 57).

### 3.2 Primary and Secondary Outcomes

Post‐operative median IV morphine consumption within the first 3 h was 5 mg (0–15) in the clonidine group and 10 mg (0–15) in the placebo group (*p* = 0.58). Similarly, no difference was observed in IV morphine consumption during the first 6 h: 6.7 mg (3.3–19.0) with clonidine and 10.0 mg (1.7–16.6) with placebo (*p* = 0.76).

**FIGURE 2:**
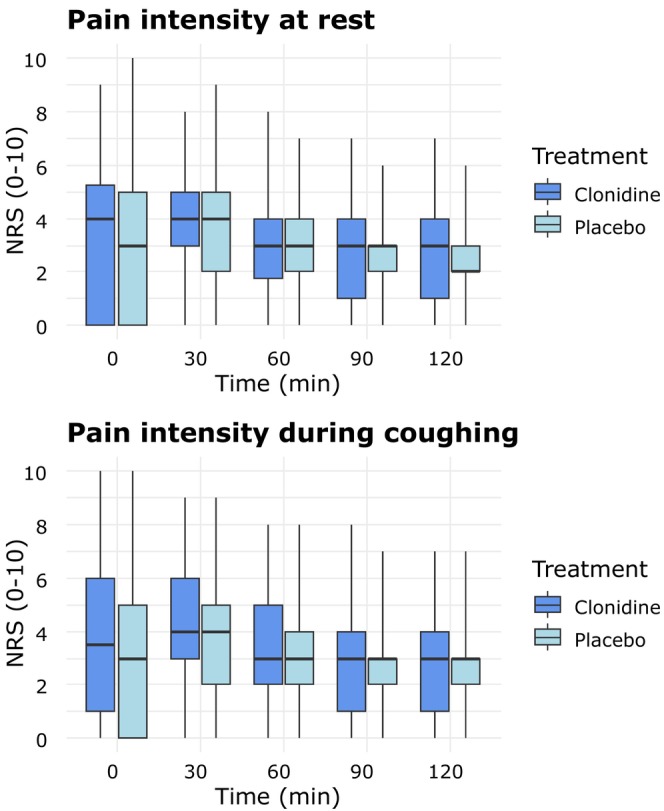
Pain intensity at rest and pain intensity during coughing. NRS, numeric rating scale. The median is represented by the bold horizontal line within the box. The box defines the interquartile range (IQR), with the upper and lower whiskers defining the minimum and maximum values, respectively.

Pain intensity (NRS, 0–10) at rest and during coughing was similar between the clonidine and placebo groups at all time points (Figure 2). The median PACU time was 105 min (90–134) for clonidine and 120 min (90–138) for placebo (*p* = 0.46). The median hospital stay was 25 h (22–31) for clonidine and 26 h (22–43) for placebo (*p* = 0.56). At 1 month, 13 clonidine patients and 9 placebo patients reported daily opioid use. The median oral morphine consumption was similar: 30 (10–30) mg in the clonidine group versus 20 mg (15–30) in the placebo group (*p* = 0.39).

### 3.3 Side Effects and Severe Adverse Events

PONV occurrence and sedation levels were similar between the clonidine and placebo groups during the PACU observation period (Table 3). At PACU arrival, 7 clonidine patients and 13 placebo patients had a Ramsay Sedation Scale > 2 (Table 3). Hypotension was treated more frequently with clonidine (25 vs. 13 patients, *p* = 0.04). Bradycardia was treated in two patients in the clonidine group and one patient in the placebo group, with no cases of arrhythmia in either group. One patient in the placebo group developed severe respiratory arrest upon PACU arrival, which was successfully treated with naloxone.

**TABLE 3:** Side effects in the perioperative period.

	Clonidine (*n* = 61)	Placebo (*n* = 59)	*p*
PONV (*n*)			
Arrival at the PACU	2	3	0.68
At 60 min	3	1	0.62
At discharge	1	0	1.00
Ramsay sedation scale > 2 (*n*)			
Arrival at the PACU	7	13	0.13
At 60 min	1	4	0.20
At discharge	0	1	0.49
Hypotension (*n*)	24	13	0.04
Bradycardia (*n*)	2	1	1.00

*Note:* Data are presented as numbers (*n*) and compared using the *X* ^2^ test or Fisher's exact test as appropriate. Hypotension is defined as requiring fluid therapy or vasoactive drugs, whereas bradycardia is defined as requiring atropine.

Abbreviation: PONV, postoperative nausea and vomiting.

## 4 Discussion

The main finding of this randomised trial was that a single intraoperative dose of clonidine (3 μg/kg) did not reduce post‐operative opioid consumption or pain intensity in spine surgery patients. This contrasts with previous studies suggesting that intravenous clonidine is effective in reducing both. Six comparable studies are summarised below: two evaluated a single pre‐operative intravenous dose of clonidine, while four examined a loading dose followed by continuous infusion.

Samantaray et al. [15] found that 3 μg/kg clonidine significantly reduced post‐operative fentanyl consumption and pain scores during coughing in 60 patients undergoing thoracic surgery. Similarly, Deyne et al. [27] found reduced IV piritramide consumption with 3 μg/kg clonidine in 60 patients undergoing laparoscopic abdominal surgery.

Marinangeli et al. [16] demonstrated a dose‐dependent reduction in 12‐h IV morphine consumption (5.6 ± 2.1 mg, 12.2 ± 4.2 mg, 21.4 ± 5.3 mg, 32.6 ± 9.1 mg) with three intraoperative doses of clonidine (5, 3 and 2 μg/kg) followed by a 12‐h post‐operative infusion (0.3 μg/kg/h) compared to placebo in 80 spine surgery patients. Post‐operative pain scores were significantly lower in the two highest‐dose groups than in the lowest dose and placebo groups. De Kock et al. [12] found significantly lowered 12‐h IV morphine consumption in the clonidine group (4 μg/kg loading dose, 2 μg/kg/h infusion) compared to placebo (19.7 ± 11.1 vs. 27.6 ± 18.1 mg) in 200 patients undergoing major abdominal surgery. However, the anaesthetic staff was not blinded to treatment allocation. Similarly, Bernard et al. [13] reported significantly lower intramuscular morphine consumption in the clonidine group (5 μg/kg loading dose, 0.3 μg/kg/h infusion) than in the placebo group in 50 patients undergoing instrumented spinal fusion. In contrast, Striebel et al. reported no significant difference in post‐operative meperidine use or pain intensity between patients receiving clonidine (150 μg loading dose, 150 μg infusion) and placebo in 60 women undergoing cholecystectomy, aligning with our study's negative findings [26].

Recent RCTs have explored the perioperative effects of intravenous clonidine on post‐operative analgesia and haemodynamic stability. Patel et al. [18] reported reduced pain and analgesic requirements with clonidine (1 μg/kg) combined with ketamine (1 mg/kg) but not clonidine alone. Gupta et al. [20] found similar pain intensities between intraperitoneal and intravenous clonidine (3 μg/kg) after hysterectomy. Mohamed et al. [19] observed significantly lower post‐operative analgesic consumption with intravenous clonidine (1.5 μg/kg) compared to placebo after laparoscopic cholecystectomy. Similarly, Silva et al. [21] observed reduced morphine use with intravenous (2 μg/kg) or intrathecal clonidine (1 μg/kg) compared to placebo for the same procedure.

The lack of effect of clonidine in our study may be due to several factors. First, the dose may have been too low to achieve an analgesic effect. We chose 3 μg/kg to balance analgesic efficacy with minimal side effects, as a higher loading dose (5 μg/kg) was associated with more frequent side effects, including hypotension and sedation [16]. Second, the absence of continuous infusion might have limited its effect. Third, the short monitoring period for post‐operative opioid consumption may explain our results. Measuring opioid consumption during the first 12 h might have revealed an effect. Fourth, the use of multimodal pain management (paracetamol, fentanyl, ketorolac, local infiltration analgesia, and morphine or oxycodone) in both groups likely reduced post‐operative pain and opioid requirements, potentially masking any additional benefit of clonidine. While this approach enhances the external validity of our study by making it more comparable to clinical practice, it also makes detecting differences between groups more challenging. Fifth, studying another population than spine surgery patients may have revealed an effect. However, spine surgery patients are at risk of moderate to severe post‐operative pain and prolonged opioid use, and a pre‐trial audit showed IV morphine consumption of 15–45 mg in the first 3 h. Unfortunately, the placebo group in our study had much lower opioid consumption, limiting the detection of a significant reduction. On the other hand, pain intensity at rest in the placebo group was 3 (0–5) upon PACU arrival and 4 [2, 3, 4, 5] after 30 min, suggesting that this patient group could have allowed detection of a significant difference in pain intensity.

In our study, more patients in the clonidine group were treated for hypotension, but the PACU stay was not prolonged in this group. Most previous studies did not report increased treatment‐requiring hypotension [12, 15, 18, 27, 28]. This discrepancy may be due to our inclusion of patients over 70 years and those with an ASA classification greater than 2.

### 4.1 Strengths and Limitations

The study's strength includes its blinded, placebo‐controlled design and single‐centre setting, ensuring consistent procedures in anaesthesia, surgery and post‐operative pain management. Successful randomisation minimised selection bias and enhanced internal validity.

The study also has some limitations. First, our power calculation was based on a 10 mg reduction in IV morphine consumption, but this difference could not be demonstrated, as the placebo group's mean morphine consumption was already below 10 mg. Second, we did not use a patient‐reported outcome measure (PROM) as the primary outcome, such as pain intensity, patient satisfaction or quality of life. However, pain intensity was included as a secondary outcome. Third, it can be argued that a more accurate measurement of opioid requirement could have been obtained using a patient‐controlled analgesia device. Last, there may have been a risk of unblinding if bradycardia and hypotension were exclusive to the clonidine group. However, treatment‐requiring hypotension occurred in both groups, and only three patients experienced bradycardia, making unblinding less likely.

## 5 Conclusion

In this randomised, double‐blinded, placebo‐controlled trial, intraoperative clonidine did not reduce post‐operative opioid consumption or pain intensity in patients undergoing spine surgery.

## Author Contributions

S.B., P.G.U., and L.N. initiated the trial. All authors contributed to study design, protocol development, and manuscript preparation. All authors read and approved the final manuscript. L.N. served as the sponsor and S.B. as the primary investigator.

## Conflicts of Interest

The authors declare no conflicts of interest.

## Data Availability

Data supporting this study's findings are available from the corresponding author upon request but are not publicly available due to privacy or ethical restrictions.

## References

[1] A. M. Rasmussen , M. H. Toft , H. N. Awada , et al., “Waking Up in Pain: A Prospective Unselected Cohort Study of Pain in 3702 Patients Immediately After Surgery in the Danish Realm,” Regional Anesthesia and Pain Medicine 46, no. 11 (2021): 948–953. 34408068 10.1136/rapm-2021-102583

[2] J. Katz and Z. Seltzer , “Transition From Acute to Chronic Postsurgical Pain: Risk Factors and Protective Fa,” Expert Review of Neurotherapeutics 9, no. 5 (2009): 723–744. 19402781 10.1586/ern.09.20

[3] H. Kehlet , T. S. Jensen , and C. J. Woolf , “Persistent Postsurgical Pain: Risk Factors and Prevention,” Lancet 367, no. 9522 (2006): 1618–1625. 16698416 10.1016/S0140-6736(06)68700-X

[4] P. Lavand'homme , “Transition From Acute to Chronic Pain After Surgery,” Pain 158, no. Suppl 1 (2017): S50–S54. 28134653 10.1097/j.pain.0000000000000809

[5] P. Uhrbrand , P. Helmig , S. Haroutounian , S. T. Vistisen , and L. Nikolajsen , “Persistent Opioid Use After Spine Surgery: A Prospective Cohort Study,” Spine 46, no. 20 (2021): 1428–1435. 34559754 10.1097/BRS.0000000000004039

[6] L. Nikolajsen and S. Haroutiunian , “Intravenous Patient‐Controlled Analgesia for Acute Postoperative Pain,” European Journal of Pain Supplements 5, no. 2 (2011): 453–456.

[7] M. D. Neuman , B. T. Bateman , and H. Wunsch , “Inappropriate Opioid Prescription After Surgery,” Lancet 393, no. 10180 (2019): 1547–1557. 30983590 10.1016/S0140-6736(19)30428-3 PMC6556783

[8] M. C. Sanchez Munoz , M. De Kock , and P. Forget , “What Is the Place of Clonidine in Anesthesia? Systematic Review and Meta‐Analyses of Randomized Controlled Trials,” Journal of Clinical Anesthesia 38 (2017): 140–153. 28372656 10.1016/j.jclinane.2017.02.003

[9] T. L. Yaksh , “Pharmacology of Spinal Adrenergic Systems Which Modulate Spinal Nociceptive Processing,” Pharmacology, Biochemistry, and Behavior 22, no. 5 (1985): 845–858. 2861606 10.1016/0091-3057(85)90537-4

[10] S. Jamadarkhana and S. Gopal , “Clonidine in Adults as a Sedative Agent in the Intensive Care Unit,” Journal of Anaesthesiology Clinical Pharmacology 26, no. 4 (2010): 439–445, 10.4103/0970-9185.74581. 21547166 PMC3087273

[11] A. Keränen , S. Nykänen , and J. Taskinen , “Pharmacokinetics and Side‐Effects of Clonidine,” European Journal of Clinical Pharmacology 13, no. 2 (1978): 97–101. 658114 10.1007/BF00609752

[12] M. F. De Kock , G. Pichon , and J. L. Scholtes , “Intraoperative Clonidine Enhances Postoperative Morphine Patient‐Controlled Analgesia,” Canadian Journal of Anaesthesia 39, no. 6 (1992): 537–544, 10.1007/BF03008314. 1643675

[13] J. M. Bernard , J. L. Hommeril , N. Passuti , and M. Pinaud , “Postoperative Analgesia by Intravenous Clonidine,” Anesthesiology 75, no. 4 (1991): 577–582, 10.1097/00000542-199110000-00006. 1928767

[14] J. W. Flacke , “Alpha 2‐Adrenergic Agonists in Cardiovascular Anesthesia,” Journal of Cardiothoracic and Vascular Anesthesia 6, no. 3 (1992): 344–359, 10.1016/1053-0770(92)90156-2. 1351752

[15] A. Samantaray , M. H. Rao , and A. Chandra , “The Effect on Post‐Operative Pain of Intravenous Clonidine Given Before Induction of Anaesthesia,” Indian Journal of Anaesthesia 56, no. 4 (2012): 359–364. 23087458 10.4103/0019-5049.100817 PMC3469914

[16] F. Marinangeli , A. Ciccozzi , F. Donatelli , et al., “Clonidine for Treatment of Postoperative Pain: A Dose‐Finding Study,” European Journal of Pain 6, no. 1 (2002): 35–42. 10.1053/eujp.2001.0270 11888226

[17] Z. M. Naja , R. Khatib , F. M. Ziade , et al., “Effect of Clonidine Versus Dexmedetomidine on Pain Control After Laparoscopic Gastric Sleeve: A Prospective, Randomized, Double‐Blinded Study,” Saudi Journal of Anaesthesia 8, no. Suppl 1 (2014): S57–S62. 25538523 10.4103/1658-354X.144078 PMC4268530

[18] J. Patel , R. Thosani , J. Kothari , P. Garg , and H. Pandya , “Clonidine and Ketamine for Stable Hemodynamics in Off‐Pump Coronary Artery Bypass,” Asian Cardiovascular and Thoracic Annals 24, no. 7 (2016): 638–646. 27471314 10.1177/0218492316663359

[19] H. S. Mohamed Ali , G. S. Gad , and H. M. Fayed , “A Comparative Study of Clonidine and Magnesium Sulfate Premedication on Perioperative Hormonal Stress Responses, Hemodynamic Stability and Postoperative Analgesia in Patients With Gallbladder Diseases Undergoing Laparoscopic Cholecystectomy. A Randomized, Double‐Blind, Controlled Study,” Egyptian Journal of Anaesthesia 38, no. 1 (2022): 108–115.

[20] D. Gupta , P. Mangwana , R. Sharma , B. Wadhwa , and S. Kerai , “Intravenous Clonidine Versus Intraperitoneal Clonidine for Postoperative Analgesia After Total Abdominal Hysterectomy: A Randomised Controlled Trial,” Turkish Journal of Anaesthesiology and Reanimation 49, no. 2 (2021): 118–123, 10.5152/TJAR.2020.55938. 33997840 PMC8098739

[21] C. R. D. Silva , C. Oliveira , and J. C. Nunes , “Comparison Between Intravenous and Intrathecal Clonidine for Postoperative Analgesia of Patients Submitted to Laparoscopic Cholecystectomy: Randomized Clinical Trial,” Brazilian Journal of Anesthesiology 72, no. 1 (2022): 135–141. 34119566 10.1016/j.bjane.2021.03.029 PMC9373681

[22] S. Birkebæk , L. M. Lundsgaard , N. Juul , et al., “Intraoperative Clonidine in Endometriosis and Spine Surgery: A Protocol for Two Randomised, Blinded, Placebo‐Controlled Trials,” Acta Anaesthesiologica Scandinavica 68, no. 5 (2024): 708–713. 38462487 10.1111/aas.14398

[23] P. Fritzell , O. Hägg , P. Wessberg , and A. Nordwall , “Chronic Low Back Pain and Fusion: A Comparison of Three Surgical Techniques: A Prospective Multicenter Randomized Study From the Swedish Lumbar Spine Study Group,” Spine 27, no. 11 (2002): 1131–1141. 12045508 10.1097/00007632-200206010-00002

[24] B. I. Martin , S. K. Mirza , N. Spina , W. R. Spiker , B. Lawrence , and D. S. Brodke , “Trends in Lumbar Fusion Procedure Rates and Associated Hospital Costs for Degenerative Spinal Diseases in the United States, 2004 to 2015,” Spine 44, no. 5 (2019): 369–376. 30074971 10.1097/BRS.0000000000002822

[25] S. Nielsen , L. Degenhardt , B. Hoban , and N. Gisev , “A Synthesis of Oral Morphine Equivalents (OME) for Opioid Utilisation Studies,” Pharmacoepidemiology and Drug Safety 25, no. 6 (2016): 733–737. 26693665 10.1002/pds.3945

[26] P. A. Harris , R. Taylor , R. Thielke , J. Payne , N. Gonzalez , and J. G. Conde , “Research Electronic Data Capture (REDCap)–A Metadata‐Driven Methodology and Workflow Process for Providing Translational Research Informatics Support,” Journal of Biomedical Informatics 42, no. 2 (2009): 377–381. 18929686 10.1016/j.jbi.2008.08.010 PMC2700030

[27] C. De yne , M. Struys , R. Heylen , et al., “Influence of Intravenous Clonidine Pretreatment on Anesthetic Requirements During Bispectral EEG‐Guided Sevoflurane Anesthesia,” Journal of Clinical Anesthesia 12, no. 1 (2000): 52–57, 10.1016/S0952-8180(99)00138-5. 10773509

[28] W. H. Striebel , D. I. Koenigs , and J. A. Krämer , “Intravenous Clonidine Fails to Reduce Postoperative Meperidine Requirements,” Journal of Clinical Anesthesia 5, no. 3 (1993): 221–225. 8318241 10.1016/0952-8180(93)90019-b

